# Screening differentially expressed genes of pancreatic cancer between Mongolian and Han people using bioinformatics technology

**DOI:** 10.1186/s12885-020-06722-7

**Published:** 2020-04-09

**Authors:** Jiasheng Xu, Kaili Liao, Zhonghua Fu, Zhenfang Xiong

**Affiliations:** 1 Department of Pathology, the First Affiliated Hospital of Nanchang University, No.17 YONGWAIZHENG Street, Nanchang, 330006 Jiangxi China; 2 Department of Clinical Laboratory, the Second Affiliated Hospital of Nanchang University, No. 1 Minde Road, Nanchang, 330006 Jiangxi China; 3 Department of Burns, the First Affiliated Hospital of Nanchang University, No.17 YONGWAIZHENG Street, Nanchang, 330006 Jiangxi China

**Keywords:** Pancreatic ductal cell carcinoma, Affymetrix gene expression profile, Gene differential expression, GO analysis, Pathway analysis, Mongolian

## Abstract

**Background:**

To screen and analyze differentially expressed genes in pancreatic carcinoma tissues taken from Mongolian and Han patients by Affymetrix Genechip. Methods: Pancreatic ductal cell carcinoma tissues were collected from the Mongolian and Han patients undergoing resection in the Second Affiliated Hospital of Nanchang University from March 2015 to May 2018 and the total RNA was extracted. Differentially expressed genes were selected from the total RNA qualified by Nanodrop 2000 and Agilent 2100 using Affymetrix and a cartogram was drawn; The gene ontology (GO) analysis and Pathway analysis were used for the collection and analysis of biological information of these differentially expressed genes. Finally, some differentially expressed genes were verified by real-time PCR.

**Results:**

Through the microarray analysis of gene expression, 970 differentially expressed genes were detected by comparing pancreatic cancer tissue samples between Mongolian and Han patients. A total of 257 genes were significantly up-regulated in pancreatic cancer tissue samples in Mongolian patients; while a total of 713 genes were down-regulated. In the Gene Ontology database, 815 differentially expressed genes were identified with clear GO classification, and CPB1 gene showed the highest increase in expression level (multiple difference: 31.76). The pathway analysis detected 28 signaling pathways that included these differentially expressed genes, involving a total of 178 genes. Among these pathways, the enrichment of differentially expressed genes in the FAK signaling pathway was the strongest and COL11A1 gene showed the highest multiple difference (multiple difference: 5.02). The expression of differentially expressed genes CPB1, COL11A1、ITGA4、BIRC3、PAK4、CPA1、CLPS、PIK3CG and HLA-DPA1 determined by real-time PCR were consistent with the results of gene microarray analysis.

**Conclusions:**

The results of microarray analysis of gene expression profiles showed that there are a large number of differentially expressed genes in pancreatic cancer tissue samples comparing Mongolian and Han population. These genes are closely related to the cell proliferation, differentiation, invasion, metastasis and multi-drug resistance in pancreatic cancer. They are also involved in the regulation of multiple important signaling pathways in organisms.

## Introduction

Pancreatic cancer has the highest mortality among all the digestive system malignancies. Its onset is occult, followed by rapid progress, so most patients are diagnosed late and lost the best treatment opportunities. Therefore, the search for specific and sensitive early diagnostic tools for the pancreatic cancer is particularly important to improve the prognosis of patients with pancreatic cancer and prolong their survival time [1]. Recent years, the completion of Human Genome Project and the maturation in gene microarray technology provide a large amount of information for high-throughput analysis of the occurrence and development of tumors, making genetic diagnosis an ideal early diagnostic method for most tumors. By analyzing genes involved in the generation and maintaining of malignant biological characteristics of pancreatic cancer, this method [2] can elucidate the molecular mechanism of pancreatic cancer. In this study, Affymetrix Gene Expression Profiling microarray was used to screen differentially expressed genes in pancreatic cancer samples of Mongolian and Han patients for further gene function analysis, in order to provide important reference data and experimental evidence for the elucidation of the molecular mechanism of pancreatic cancer development.

## Methods

### Reagents and instruments

The required reagents Agilent RNA 6000 Nano Kit, GeneChip 3’IVT Express Kit, GeneChip Hybridization Wash and Stain Kit, Trizol Kit, and QIAGEN RNeasy Total RNA Isolation kit were provided by Gekkai Gene Technology Co., Ltd. The main instruments used in the study included the Thremo Nanodrop 2000, Agilent 2100, GeneChip Hybridization Oven 645, GeneChip Fluidics Station 450, and GeneChip Scanner 3000.

### Sample information

A total of 96 fresh tumor tissue specimens from patients with pancreatic malignancies surgically resected in the Second Affiliated Hospital of Nanchang University and the First Affiliated Hospital of Nanchang University from March 2015 to May 2018 were collected. 66 of them were initially selected according to the following inclusion and exclusion criteria and these specimens were used for follow-up gene expression microarray assays.

#### Inclusion criteria

(1) Pathologically confirmed pancreatic ductal cell carcinoma;

(2) New cases diagnosed by our hospital for the first time; (3) Patients aged ≥18 years; (4) Patients and their families agree to provide tissue specimens for scientific research and allow the publication of research data.

#### Exclusion criteria

(1) Pathologically confirmed local vascular invasion and regional lymph node metastasis; (2) distant metastasis found by relevant examinations or during operation in our hospital; (3) with other malignant tumors or diabetes, hypertension, systemic diseases such as cardiovascular or cerebrovascular diseases; (4) patients who have received radiotherapy, chemotherapy, or other anti-neoplastic drugs before surgery; (5) with a history of surgery in the past 3 years.

#### General information of patients

Of the 66 patients, 32 were Mongolian and 34 were Han; 34 were males and 32 were females; the average age was 60.12; the youngest patient was 42 years-old and the oldest was 73 years-old; 24 cases were well-differentiated, 38 cases were moderately differentiated, and 4 cases were poorly differentiated.

### Sample Total RNA extraction and quality assurance

The collected samples were extracted and purified by Trizol kit and QIAGEN RNeasy Total RNA Isolation kit to obtain the total RNA. The samples were tested using Nanodrop 2000 and Agilent 2100 instruments and evaluated according to RNA concentration, A260/A280 value, RIN value and 28S/18S value. When the A260/A280 was between 1.7 and 2.2, RIN value was smaller than A26 and 28S/18S value were bigger than 0.7, the samples were qualified. The above procedures were strictly carried out in line with the reagent and instrument instructions.

### Gene microarray hybridization

The double-stranded cDNA template was synthesized after mixing the purified total RNA with the internal reference poly-A RNA according to the instructions of the GeneChip 3′ IVT Express Kit. Then, the biotin-labeled amplified RNA (aRNA) was obtained by reverse transcription in vitro. The obtained aRNA was purified using the purification reagent in the kit and fragmented to prepare a hybridization reaction solution. The hybridization reaction solution was heated at the 98 °C waterbath for 10 min until the temperature of solution rose up to 45 °C. Then the waterbath was retained at 45 °C for more than 3 mins. Meanwhile, 130 μL of the pre-hybridization solution in the kit was injected into the chip and kept in the hybridization oven at 45 °C for 10 min. After that, the pre-hybridization solution was discarded, and 130 μL of the hybridization reaction solution was injected into the chip at 45 °C and the hybridization was performed at 60 rpm for 16 h to complete the hybridization. Afterwards, automatic washing and dyeing was performed using the GeneChip Fluidics Station 450 instrument, then the chip was scanned to obtain data.

### Real-time quantitative PCR verification

The top 10 differentially expressed genes in Han and Mongolian pancreatic cancer patients were selected and verified. Primers were synthesized according to the PCR primer information provided by the Primer Bank database (Table 1). GAPDH was used as an internal reference and a two-step method was used. The expression of GAPDH was detected by qPCR. Using the expression level of GAPDH as the standard value “1”, the relative expression levels of each differential gene in Mongolian and Han pancreatic cancer tissues and adjacent tissues were calculated. The real-time PCR kit was used to detect the expression of these genes in pancreatic cancer tissues and their adjacent normal tissues and to draw statistical charts. The reaction procedure was: Hol d (pre-denaturation): 95 °C, 30 s, 1 cycle; Two-step PCR: 95 °C, 5 s, 60 °C, 30 s, 40 cycles; Dissociation: 95 °C, 15 s, 60 °C, 30 s, 95 °C, 15 s, 1 cycle.

**Table 1:** Gene primers for real-time PCR

Gene Symbol	Gene Id	PrimerBank ID	Amplicon Size	Forward Primer	Reverse Primer
CPB1	1360	54607079c1	194	CCAGCACGACCCAGATTGAC	AACCCGGCTATCAAACTGAGC
ITGA4	3676	67191026c1	139	CACAACACGCTGTTCGGCTA	CGATCCTGCATCTGTAAATCGC
BIRC3	330	342307084c1	79	AAGCTACCTCTCAGCCTACTTT	CCACTGTTTTCTGTACCCGGA
PAK4	10,298	126273532c1	113	GGACATCAAGAGCGACTCGAT	CGACCAGCGACTTCCTTCG
COL11A1	1301	98985805c1	128	ACCCTCGCATTGACCTTCC	TTTGTGCAAAATCCCGTTGTTT
GAPDH	26,330	126273608c1	116	TGTGGGCATCAATGGATTTGG	ACACCATGTATTCCGGGTCAAT
CPA1	1357	221316610c1	82	GCTGGTGTTGAGTGTCCTGTT	GGCTACAGAGATTCGGAGCAC
CLPS	389,383	155029555c1	92	GCGCTTCCGCAATATAAAAAGAA	CCGGAGTCCAAGTCCATGAG
PIK3CG	5294	21237724c1	150	GGCGAAACGCCCATCAAAAA	GACTCCCGTGCAGTCATCC
HLA-DPA1	3113	335883184c1	112	ATGCGCCCTGAAGACAGAATG	ACACATGGTCCGCCTTGATG

### Data processing and statistical analysis

The data obtained by scanning was analyzed using the R-Project software in line with the following protocol:

Filtering the background noise with the 20% lowest signal strength among all microarray probes;
Using the software to plot the signal intensity distribution of the probes and the quadrantal diagram of the relative logarithm of the probe signal intensity, in order to evaluate the reliability and repeatability of the results of the differential expression profile microarray;
(3) Gene differential expression analysis was performed using the GCBI online analysis tool (https://www.gcbi.com.cn/gclib/html/index). GCBI (Gene-Cloud of Biotechnology Information) is a R-based online analysis software developed in China, which can conveniently process microarray data. Through GCBI software, the microarray data was first processed by logarithmic standard to facilitate analysis, and differential genes were screened by statistical methods. Using the linear model based on empirical Bayesian distribution to calculate the *P* value of the significant difference in gene expression between the two groups, the screening criteria for gene expression with significant differences were defined as fold change> 3.0 and *P*-value< 0.05;
Using software, scatter plots were drawn based on the signal intensity of the two sets of sample chips. The volcano plots are drawn based on the fold change and the P value of the difference test between the two groups of samples.
Hierarchical cluster analysis was used to preliminarily classify the above microarray results from two dimensions - sample and gene differential expression patterns.
Gene Ontology (GO) and Kyoto Encyclopedia of Genes and Genomes (KEGG) Pathway Analysis

The GO functional annotation refers to the description of biological functions using standard expression terms for gene and protein functions in different databases. This project was established by the Gene Ontology Consortium. The GO annotation currently includes three aspects of biological content: Biological Process, Cellular Component, and Molecular Function. This study used the DAVID online analysis website (The Database for Annotation, Visualization and Integrated Discovery, https://david.ncifcrf.gov/) to perform GO functional annotation analysis of DEGs [3]. Through GO enrichment analysis, we can clearly understand the biological function, pathway or cell location of differentially expressed genes enriched [4]. By performing GO analysis on the differentially expressed genes uploaded, the most significant differences in functions were classified into three aspects - biological process (BP), molecular function (MF) and cellular component, CC. Fisher’s fine test was used to evaluate the enrichment degree (α = 0.05) of these differentially expressed genes in each classification; KEGG (Kyoto Gene and Genome Encyclopedia, http://www.genome.jp/kegg/) was established by Kanehisa Lab at the Bioinformatics Center of Kyoto University in Japan and is based on information on genome, chemistry, and system functions. A database of biological information and biological information included in the cell, which predicts the role of proteins in various cellular activities and maps them into networks. By analyzing the differentially expressed genes in the signal pathway, one can understand the metabolic pathways that are significantly altered under disease conditions, which is of great significance for the exploration of experimental mechanisms. Based on the analysis of gene signal pathway enrichment of differentially expressed genes based on the KEGG database, the most significant differences in functions were ranked and analyzed. Both the *P* value and the false discovery rate (FDRs) ≤ 0.05 are considered to be statistically significant. By searching KEGG and BioCarta database, Fisher fine testing calculated the enrichment significance of differentially expressed genes in each signaling pathway, in order to evaluate the significantly influenced signaling pathway (a = 0.05).

## Results

### Sample total RNA quality inspection

Through total RNA quality testing, the total RNA of 55 out of 66 samples were qualified for follow-up study. 20 samples in Han patients and 20 in Mongolian patients were randomly selected. 12 male and 8 female Han patients and 11 male and 9 female Mongolian patients were selected. Among Han samples, 6 were well-differentiated, 10 were moderately differentiated and 4 were poorly differentiated; among Mongolian patients, 12 were well-differentiated, and 8 were moderately differentiated.

### Differentially expressed gene test results and quality assess ment

1. After noise reduction, 36,866 out of 49,395 probes included in the chip are selected for subsequent analysis.
2. After analysis, a total of 970 genes were differentially expressed in the above 36,866 probes, and the differential expression rate was 2.69%. Compared with the Han and Mongolian patients, the differentially expressed genes in the pancreatic cancer samples showed 257 genes were significantly up-regulated. A total of 713 genes were significantly down-regulated.
3. Data quality evaluation: The signal intensity distribution curve of each sample microarray probe fitted well, confirming the reliability of data obtained from microarray analysis.

The relative logarithm signal intensity distributions of each probe of the box plot are close to each other, confirming the repeatability of the data.

### Analysis of significant differences in sample gene expression

**Fig. 1:**
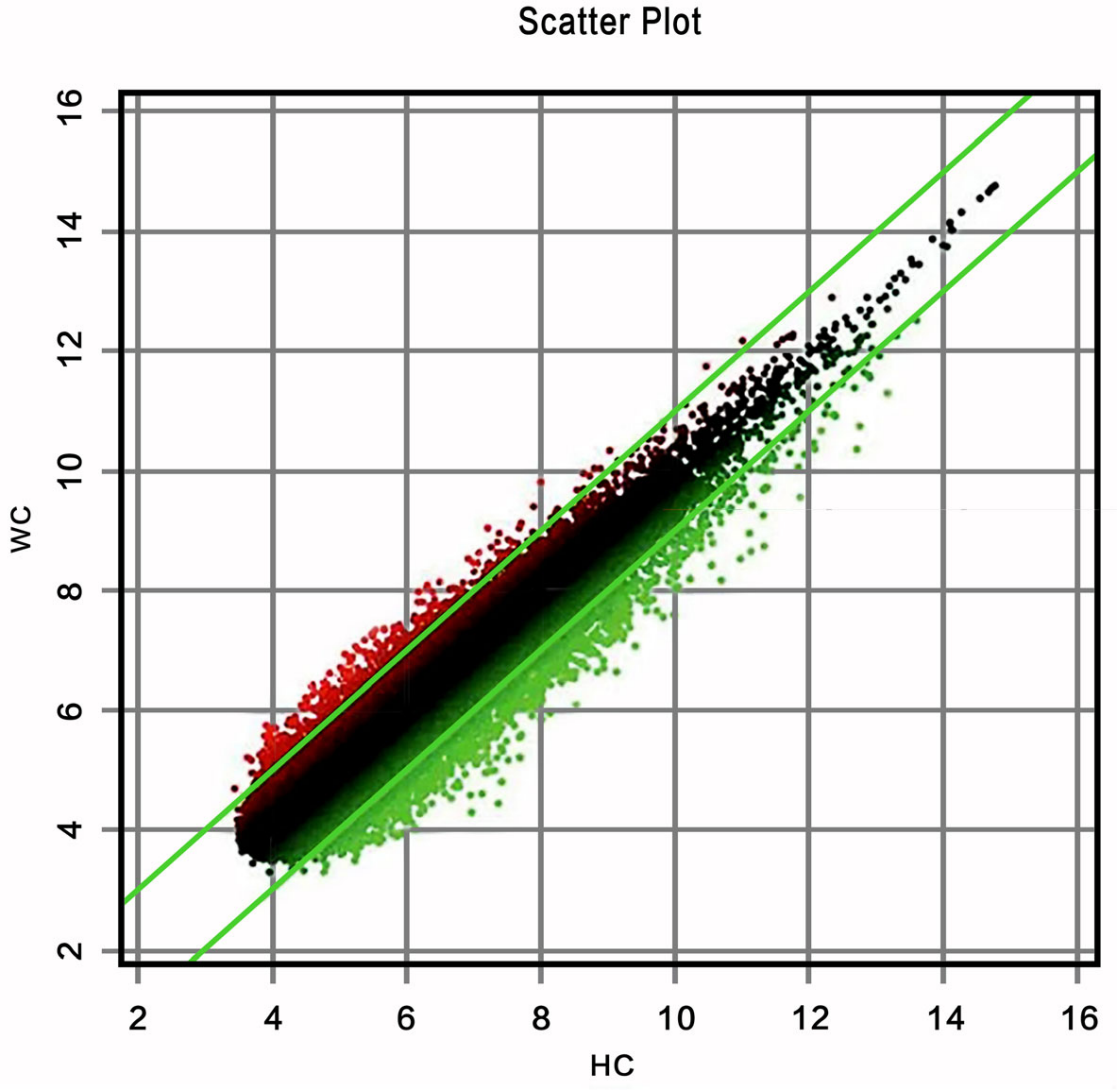
The ordinate represents the signal intensity of specimen probes from Mongolian patients with pancreatic cancer, and the abscissa represents the signal intensity of specimen probes from Han patients with pancreatic cancer. The points in the figure represent the strength of a probe in Han and Mongolian specimens. The dots within the interval between the green lines represent genes that are not significantly differently expressed. The extra-line regions are genes that are significantly differently expressed, among them the red dots indicate genes that are up-regulated, and the green dots are genes that are down-regulated

**Fig. 2:**
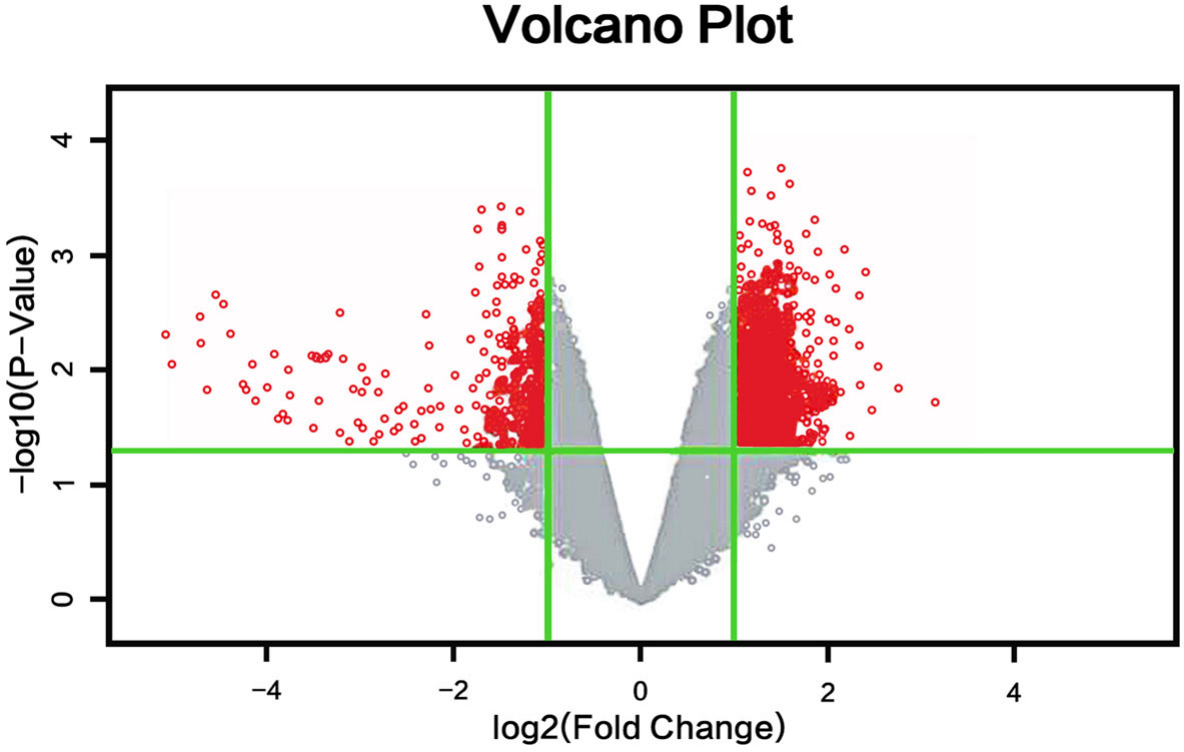
Volcanic map of differential gene. The red points indicate differentially expressed genes that have statistically significant. The grey points indicate differentially expressed genes that have no statistically significant

**Fig. 3:**
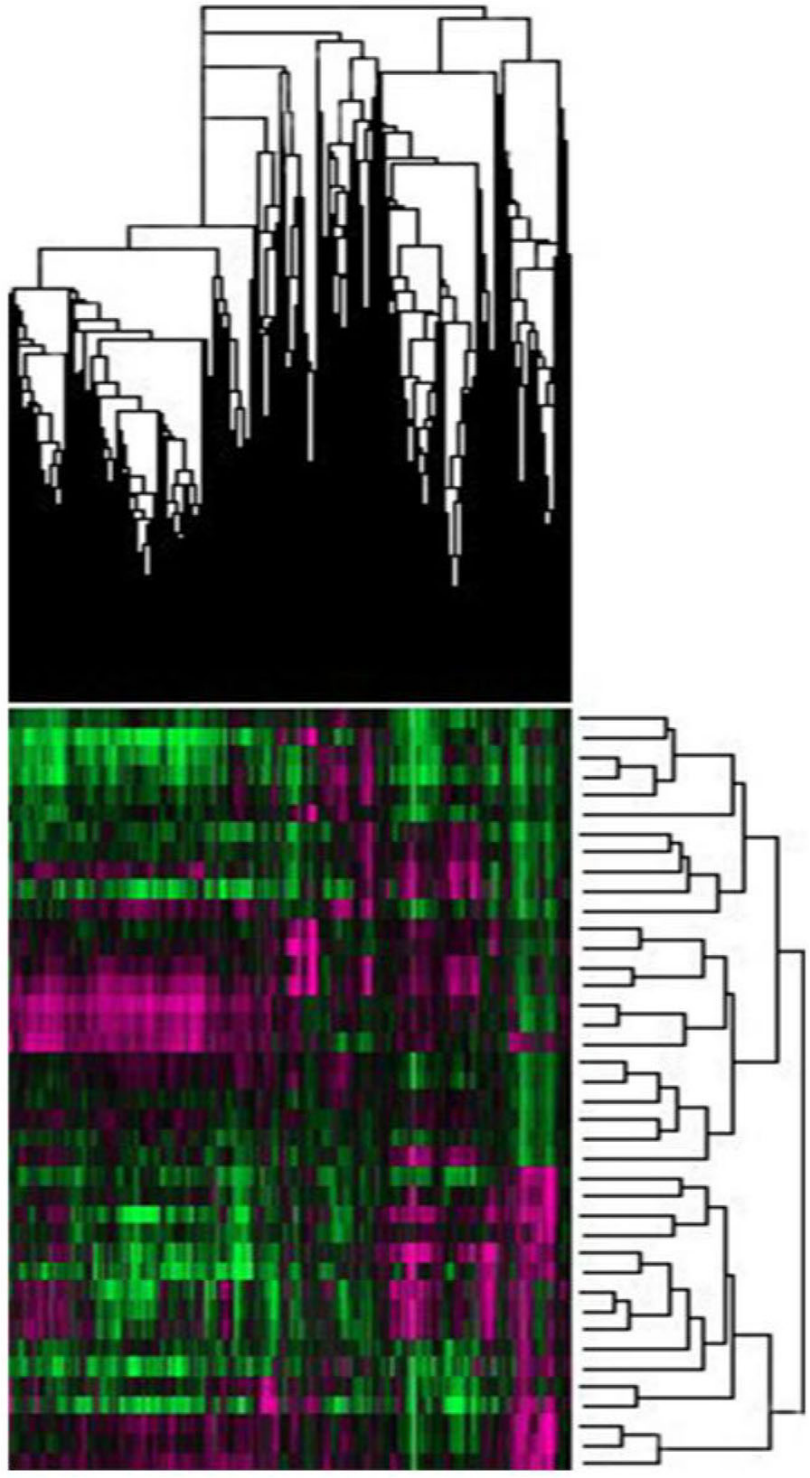
Hierarchical cluster analysis hotmap: The result of cluster analysis of the significantly differently expressed gene expression profiles between two groups of samples. The columns in the figure represent the samples and the rows represent the differentially expressed genes

1. The scatter plot is shown in Fig. 1. The ordinate represents the signal intensity of specimen probes from Mongolian patients with pancreatic cancer, and the abscissa represents the signal intensity of specimen probes from Han patients with pancreatic cancer. The points in the figure represent the strength of a probe in Han and Mongolian specimens. The dots within the interval between the green lines represent genes that are not significantly differently expressed. The extra-line regions are genes that are significantly differently expressed, among them the red dots indicate genes that are up-regulated, and the green dots are genes that are down-regulated.
2. Figure 2 is a volcano plot drawn by the software based on the gene expression differential multiples and the *P* value of the significance test. The ordinate is the P value, and the abscissa is the logarithmic transformed difference multiplier value (base = 2). The red points indicate differentially expressed genes that meet the above significant differential expression screening conditions.
3. Figure 3 is a hotspot plot of hierarchical cluster analysis showing the gene expression profiles of the two groups with significant expression level differences between the two samples. The columns represent samples and the rows represent differentially expressed genes. The results show that most of the samples in the same ethnic group have similar differential gene expression profiles; according to the left-hand dendrogram in the figure, some genes have similar expression patterns. These genes may have similar functions or participate in the same biological process. Genes with the highest differential expression times were named as a gene cluster. For example, the RASA2 gene cluster includes BIRC2, RASA2, ADAM17, RECQL, LYAR, and SDHD genes, of which the differential expression of RASA2 is the highest (2.36 times).

### Differentially expressed genes bioinformatics analysis

**Fig. 4:**
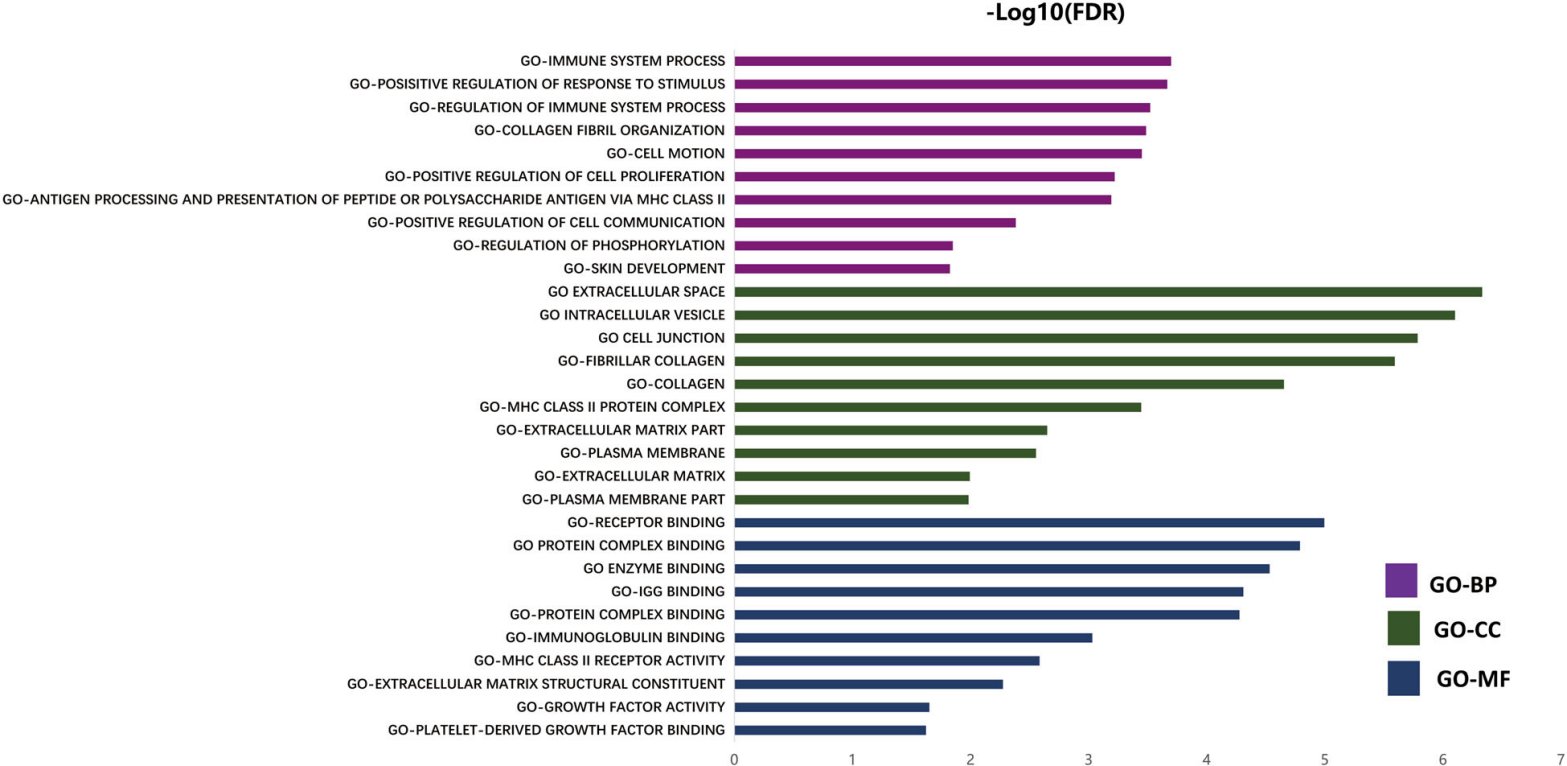
Gene Ontology analysis shows the top 10 GO categories classified according to the level of differentially expressed genes enriched in the three major classifications

**Fig. 5:**
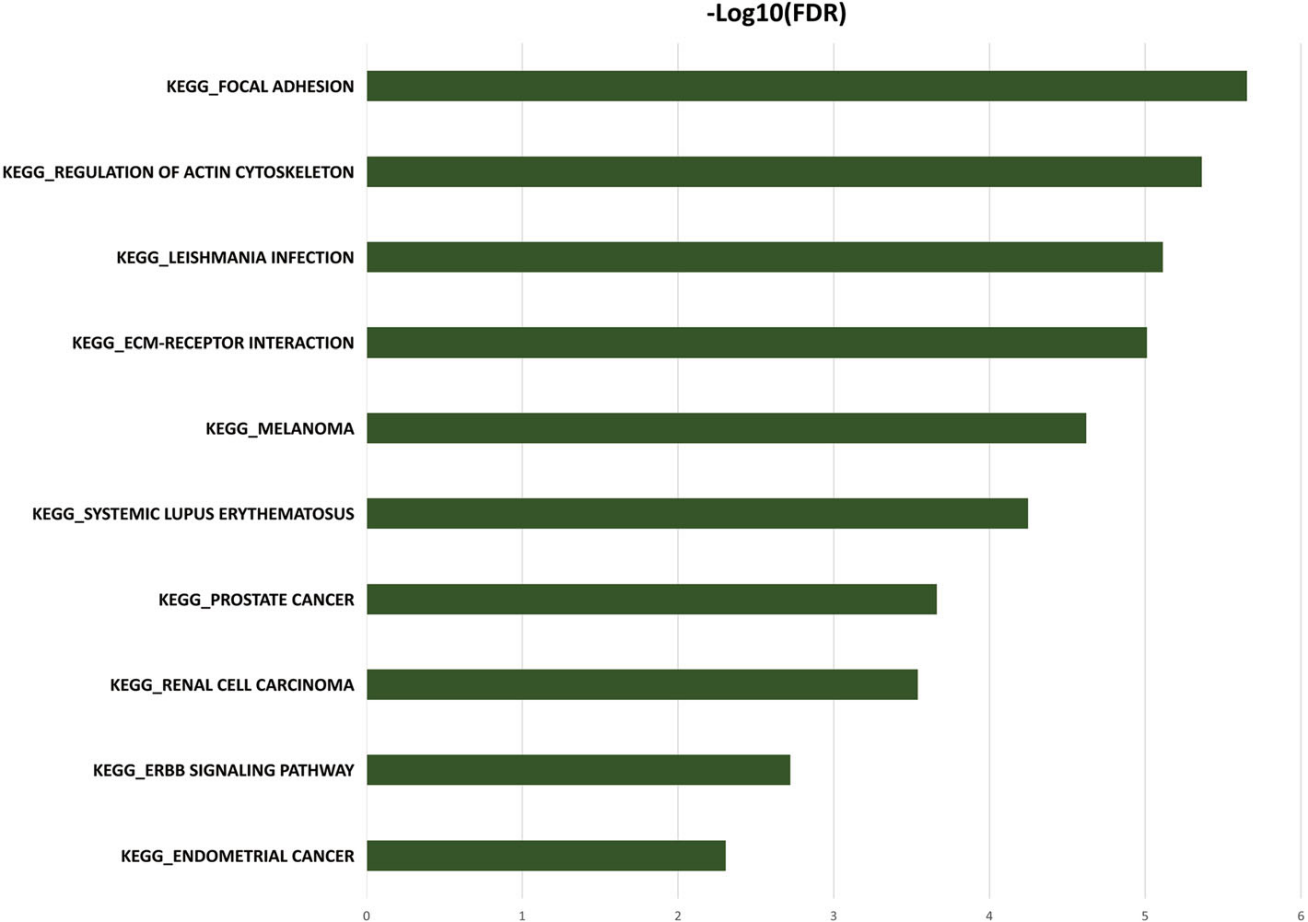
Pathway analysis shows the total of 28 cellular signaling pathways were differentially expressed between the two groups of samples

1. GO analysis: Based on the results of the differentially-expressed gene screening, 793 differentially expressed genes were retrieved from the Gene Ontology database. Among the three major categories of GO analysis, the BP classification contains a total of 597 differentially expressed genes. In MF classification, a total of 613 genes were included. The CC classification contained a total of 573 differentially expressed genes; Fig. 4 shows the top 10 GO categories classified according to the level of differentially expressed genes enriched in the three major classifications. The results showed significant differences between the two groups of samples. Among the expressed genes, the genes involved in biological processes mainly encode proteins that control the body’s immune system, immune response, cell signal transduction, cell response to stimuli, and multiple feedback regulation; the molecular function-related genes mainly code for proteins related to endopeptidase activity, binding capacity to the same protein, enzyme regulatory activity, cytoskeletal protein binding ability, specific binding ability to protein domains, and protein molecule function regulation; the genes associated with cellular components are mainly involved in the plasma membrane structure, vacuoles, endoplasmic reticulum, cell gap junction, extracellular matrix and other structural proteins.
2. Pathway analysis: After searching KEGG and BioCarta databases, a total of 28 cellular signaling pathways were differentially expressed between the two groups of samples, involving 178 genes, as shown in Fig. 5; Tables 2, 3, and 4 are a detailed list of differentially expressed genes contained in the three strongest enriched pathways (Focal adhesion, Pathways in cancer and Regulation of actin cytoskeleton respectively).

**Table 2:** The differentially expressed genes in Focal Adhesion pathway

Gene Symbol	Gene Id	Fold Change	logFC	Regulation	P-value
ITGA4	3676	2.51	1.33	up	0.0482
BIRC3	330	3.47	1.80	up	0.0138
PAK4	10,298	−2.33	−1.22	down	0.0048
COL1A1	1277	2.24	1.16	up	0.0390
FN1	2335	2.90	1.53	up	0.0344
PDGFD	80,310	2.40	1.26	up	0.0167
COL11A	13,011	5.02	2.33	up	0.0061
PTEN	5728	2.01	1.01	up	0.0262
ITGB1	3688	2.14	1.10	up	0.0342
PIK3R2	5296	2.16	1.11	up	0.0148
COL5A1	1289	3.06	1.62	up	0.0427
PAK3	5063	−3.42	−1.77	down	0.0491
BIRC2	329	2.21	1.14	up	0.0089
PAK2	5062	2.26	1.18	up	0.0091
SHC2	25,759	−2.08	−1.06	down	0.0063
MYL12A	10,627	2.57	1.36	up	0.0050
PXN	5829	2.06	1.04	up	0.0274
PIK3R1	5295	2.30	1.20	up	0.0179
PPP1CB	5500	2.02	1.01	up	0.0124
RHOA	387	2.31	1.21	up	0.0176
EGFR	1956	3.69	1.88	up	0.0088
COL1A2	1278	3.90	1.97	up	0.0281
RAP1A	5906	2.14	1.10	up	0.0439
PDGFC	56,034	2.39	1.26	up	0.0141
CAPN2	824	2.17	1.11	up	0.0177
THBS2	7058	3.61	1.85	up	0.0340
COL6A3	1293	2.87	1.52	up	0.0326
VCL	7414	2.09	1.06	up	0.0317
CAV2	858	2.17	1.12	up	0.0363
RAP1B	5908	2.48	1.31	up	0.0162
COL3A1	1281	2.59	1.37	up	0.0397
COL5A2	1290	2.66	1.41	up	0.0498
PIK3CG	5294	2.57	1.36	up	0.0194
CCND1	595	2.12	1.09	up	0.0187
FLT1	2321	2.40	1.27	up	0.0245
PPP1R12A	4659	2.09	1.06	up	0.0083
ITGAV	3685	2.96	1.57	up	0.0036

**Table 3:** The differentially expressed genes in Regulation of actin cytoskeleton pathway

Gene Symbol	Gene Id	Fold Change	logFC	Regulation	P-value
PIK3CG	5294	2.57	1.36	up	0.0194
ARPC5	10,092	2.17	1.12	up	0.0263
ARHGEF6	9459	2.29	1.19	up	0.0376
ITGAV	3685	2.96	1.57	up	0.0036
PPP1R12A	4659	2.09	1.06	up	0.0083
VCL	7414	2.09	1.06	uup	0.0317
NCKAP1L	3071	2.04	1.03	up	0.0328
PDGFC	56,034	2.39	1.26	up	0.0141
PIK3R1	5295	2.30	1.20	up	0.0179
PXN	5829	2.06	1.04	up	0.0274
GNA13	10,672	2.65	1.41	up	0.0109
KRAS	3845	2.28	1.19	up	0.0017
TMSB4X	7114	2.54	1.34	up	0.0074
FGF7	2252	2.89	1.53	up	0.0315
EGFR	1956	3.69	1.88	up	0.0088
RHOA	387	2.31	1.21	up	0.0176
PPP1CB	5500	2.02	1.01	up	0.0124
PAK2	5062	2.26	1.18	up	0.0091
NRAS	4893	2.31	1.21	up	0.0193
ARPC3	10,094	2.08	1.06	up	0.0147
IQGAP2	10,788	2.06	1.05	up	0.0188
MYL12A	10,627	2.57	1.36	up	0.0050
ITGB1	3688	2.14	1.10	up	0.0342
PAK3	5063	−3.42	−1.77	down	0.0491
F2R	2149	2.88	1.53	up	0.0151
PIK3R2	5296	2.16	1.11	up	0.0148
MSN	4478	2.61	1.38	up	0.0190
FN1	2335	2.90	1.53	up	0.0344
PDGFD	80,310	2.40	1.26	up	0.0167
ITGA4	3676	2.51	1.33	up	0.0482
INS	3630	−3.31	−1.73	down	0.0115
PAK4	10,298	−2.33	−1.22	down	0.0048

**Table 4:** The differentially expressed genes in Leishmania infection pathway

Gene Symbol	Gene Id	Fold Change	logFC	Regulation	P-value
HLA-DPA1	3113	2.33	1.22	up	0.0373
TLR4	7099	2.05	1.03	up	0.0441
IFNGR1	3459	3.07	1.62	up	0.0165
C3	718	2.54	1.34	up	0.0274
FCGR3A	2214	3.61	1.85	up	0.0141
NCF2	4688	2.90	1.54	up	0.0030
HLA-DRA	3122	3.09	1.63	up	0.0445
HLA-DQA1	3117	2.43	1.28	up	0.0240
PTGS2	5743	3.42	1.77	up	0.0074
ITGA4	3676	2.51	1.33	up	0.0482
FCGR2A	2212	2.72	1.45	up	0.0331
FCGR2C	9103	2.72	1.45	up	0.0331
STAT1	6772	2.63	1.40	up	0.0279
HLA-DOA	3111	2.49	1.32	up	0.0466
HLA-DMB	3109	2.50	1.32	up	0.0407
ITGB1	3688	2.14	1.10	up	0.0342
HLA-DPB1	3115	2.66	1.41	up	0.0431
FCGR1A	2209	2.33	1.22	up	0.0049
FCGR3B	2215	3.61	1.85	up	0.0141
IFNGR2	3460	2.04	1.03	up	0.0271

### Real-time PCR verification

**Fig. 6:**
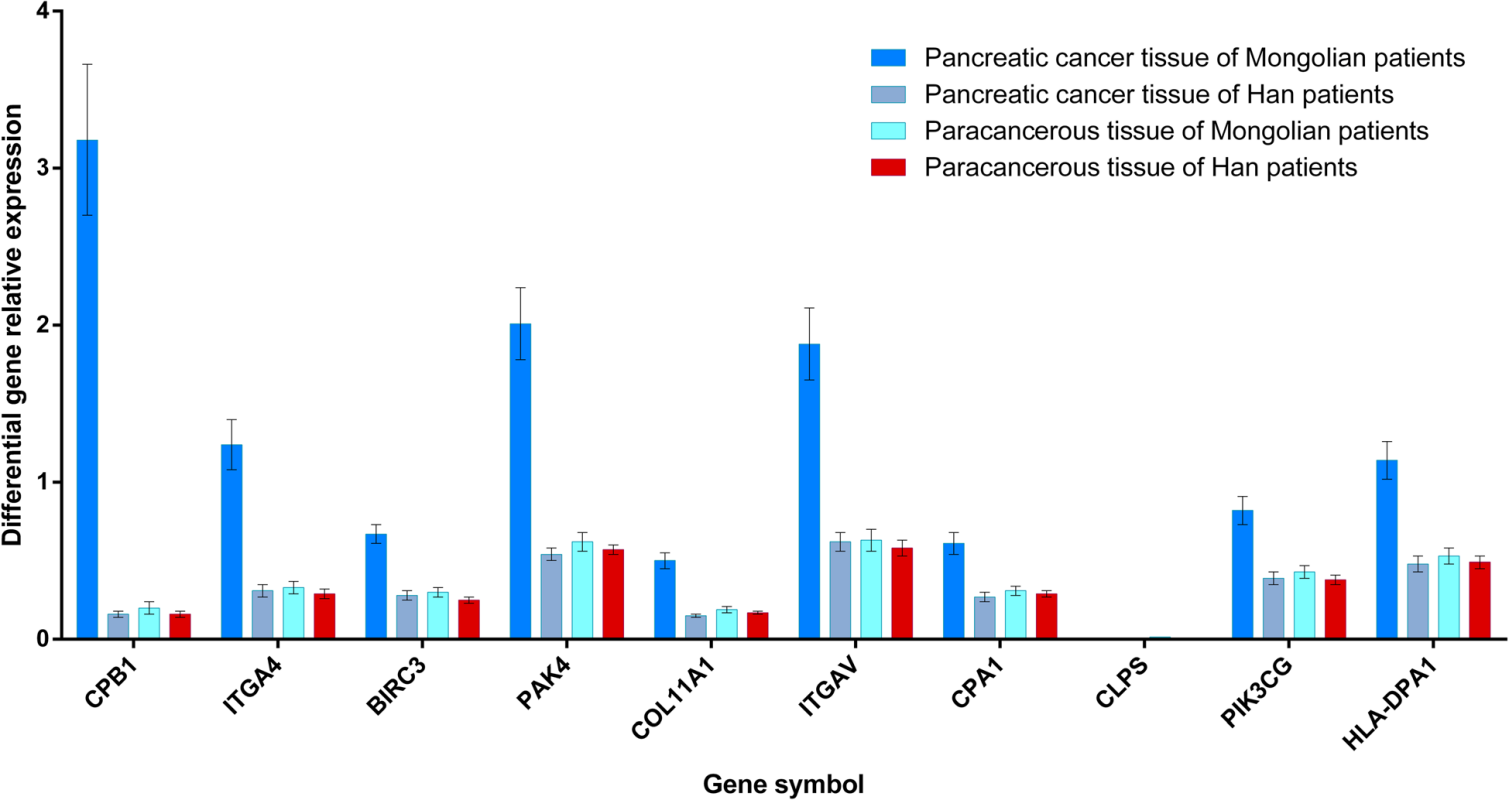
Relative expression levels of the differentially expressed genes in pancreatic cancer and normal adjacent tissues of Mongolian and Han nationalities

Results of real-time quantitative PCR detection of differentially expressed genes listed in Fig. 6, the relative expression of each gene in the figure was calculated according to the relative expression quantity = 2 - ΔCT formula, where ΔCT = CT value of target gene - CT value of internal reference gene (GAPDH), as can be seen from the figure, in addition to CLPS, the expression level of differential genes in Mongolian pancreatic cancer tissues was significantly higher than that in Han pancreatic cancer tissues. There was no significant difference in differential gene expression in pancreatic cancer adjacent tissues between Mongolian and Han patients.

## Discussion

The occurrence and development of pancreatic cancer is affected by many factors. A large number of studies have shown that the incidence of pancreatic cancer in different regions, races and even ethnic groups is significantly different. For example, Wormann SM [5] pointed out in his report that ethnic differences are one of the high-risk factors for the onset of pancreatic cancer; Investigation by Ma J [6] showed that from 1970 to 2009, the trend of the change in the mortality rate of pancreatic cancer in white and black people in the United States is diametrically opposite, suggesting that the prognosis of pancreatic cancer may be diverse between people with different genetic background [7]. At present, there are few studies on pancreatic cancer related to Mongolian population at home and abroad. There is no strong evidence to confirm that there is a significant difference in the prevalence and prognosis of pancreatic cancer between Mongolian and Han or other ethnic minorities [8].

In this study, Affymetrix gene expression microarray was used to detect and analyze differentially expressed genes and their biological information in surgically resected pancreatic cancer tissue from Mongolian and Han patients. From the molecular level we explored the potential difference in the development of pancreatic cancer in different ethnic groups. This provides a reliable reference for further elucidating the generation and maintenance of malignant biological characteristics of pancreatic cancer.

In this study, through screening differentially expressed gene in Mongolian and Han pancreatic cancer tissue samples, we found that there were 1034 genes with significant different expression level between the two groups of samples, accounting for 2.69% of the total number of detected genes. Compared to the Han patients, 257 genes were significantly up-regulated and 712 were down regulated in the Mongolian patients. According to gene ontology analysis, a total of 793 genes in the differentially expressed genes identified above were fully documented in the database. These differentially expressed genes were closely associated with the proliferation and differentiation of pancreatic cancer cells, invasion, metastasis and multidrug resistance according to the annotation of the gene biological function in the database. The highest score for differential expression and significant difference in genes associated with biological processes was the PLA2G1B (Phospholipase A2, group IB) gene, which was significantly down-regulated (9.26 times) in Mongolian pancreatic cancer tissue samples. The protein encoded by the PLA2G1B gene is phospholipase A2, which plays a key role in membrane channel activation, information transmission, hemodynamics, and pathophysiology during pancreatic inflammation and after tissue injury [3, 9]. In addition, the study by Abbenhardt C et al. [10] showed that the single nucleotide polymorphism of PLA2G1B gene is closely related to the susceptibility of rectal cancer. However, there are few reports on the association between this gene and pancreatic cancer at home and abroad. Carboxypeptidase-1 (CPB1) gene has the highest score for the differential expression and significance among all the genes related to molecular function. Compared with the Han nationality, this gene was 16.88 times down-regulated in Mongolian pancreatic cancer tissues. The CPB1 gene mainly encodes pancreatic carboxypeptidase, an important serum marker for pancreatic dysfunction [4, 11]. In recent years, there have been relatively few researches on the association between CPB1 gene and malignant tumors. Jin et al. [12] showed that CPB1 has a noticeable abnormal expression in some breast cancer patients. The study of Bouchard P et al. [13] also suggested that CPB1 may be related to the lymph node metastasis of breast cancer. The CPB1 gene may be related to the structural components in extracellular matrix of the pancreatic cancer cell. The degree of enrichment of differential genes in GO classifications can, to a certain extent, reflect the degree of differences between Mongolian and Han pancreatic cancers in the above biological classifications, but using these results alone to evaluate the differences in the biological characteristics between Mongolian and Han pancreatic cancers could be inaccurate. The distribution of the above classification in pancreatic cancer-related genes in all populations needs to be considered.

Pathway analysis is currently the most commonly used method for the analysis of gene expression microarray differential gene bioinformatics. It retrieves detailed information on biological signal transduction pathways in the two authoritative databases KEGG and BioCarta, using the pathway as a unit and using all genes included as background, to analyze and calculate the significance levels of differentially expressed genes enriched in each pathway, thereby identifying the metabolic and signal transduction pathways that were significantly affected, and clarifying the molecular regulatory mechanisms that underlie the biological function of genes. Through Pathway analysis, we detected a total of 28 signal pathways differentially expressed between Mongolian and Han pancreatic cancer tissue samples, involving a total of 178 genes, among which the FAK (Focal Adhesion Kinase) pathway was differentially expressed at the highest degree of gene enrichment. There were 37 members with significant expression differences, of which 34 were up-regulated and 3 were down-regulated. The COL11A1 gene obtained the highest score for differential expression and significant difference test. This gene was 5-time up-regulated in Mongolian pancreatic cancer tissues.

The FAK signaling pathway can integrate multiple extracellular signals to regulate the expression of downstream molecules, thereby controlling the proliferation and apoptosis of cells. This pathway is cascaded with multiple signal transduction pathways in the body and is the central link of intracellular and extracellular signal transduction [14, 15].. A large number of studies have confirmed that the abnormal activation of FAK signaling pathway is closely related to the occurrence and development of various malignant tumors [16–18]. In the study of pancreatic cancer, Gao Z et al. [19] reported that the overexpression of SRPX2 (Sushi repeat-containing protein, X-linked 2), which is dependent on the phosphorylation level of FAK, is closely related to the local invasion and distant metastasis of pancreatic cancer. The study by Hsieh YJ et al. [20] also showed that the FAK signaling pathway plays a crucial role in promoting the invasion and metastasis of pancreatic cancer cells by the newly discovered ubiquitin hydrolase family member USP22. Dao P et al. [21] pointed out in their report that intrinsic acquired drug resistance is the key factor for the clinical efficacy of a tumor necrosis factor-related apoptosis-inducing ligand (TRAIL), which is a class of potential anti-cancer drugs and is currently in the clinical research phase. Moreover, the newly discovered FAK inhibitor PH11 can induce rapid apoptosis of TRAIL-resistant PANC-1 cells, indicating that excessive activation of FAK signaling pathway may be related to multi-drug resistance of pancreatic cancer.

## Conclusion

The occurrence and development of pancreatic cancer is a complex process involving multiple factors. A large number of evidence-based medical evidence indicates that there is a significant difference in the susceptibility and incidence of pancreatic cancer in different populations. In this study, gene expression profile microarray analysis was used to screen out significant differentially expressed genes in the pancreatic cancer tissues of the Mongolian and Han populations. The functions and regulatory mechanisms of these genes were analyzed to provide a large number of genetic loci and reference data for further study on the molecular mechanisms of the generation and maintenance of malignant biological characteristics of pancreatic cancer. However, due to the limited samples of pancreatic cancer, the results are limited. There is still a need for multi-center and large sample family studies to further clarify the characteristics of the development and molecular biology of Mongolian pancreatic cancer, to design more targeted and individualized prevention and control measures, and to further promote the accurate medical treatment of patients of various ethnic group with pancreatic cancers.

## Data Availability

All datas are available. Please contact us to access if it is needed.

## Abbreviations

RNA: Ribonucleic Acid
GO: Gene Ontology
PCR: Polymerase Chain Reaction
GCBI: Gene-Cloud of Biotechnology Information
KEGG: Kyoto Encyclopedia of Genes and Genomes
DAVID: The Database for Annotation, Visualization and Integrated Discovery
DEG: Differentially Expressed Gene
BP: Biological Process
MF: Molecular Function
CC: Cellular Component
FDR: False Discovery Rate
CPB1: Carboxypeptidase-1
FAK: Focal Adhesion Kinase
